# Validity and Applicability of the Eating Motivation Survey (TEMS) in a University Population in the Western Brazilian Amazon

**DOI:** 10.3390/ijerph23010089

**Published:** 2026-01-09

**Authors:** Flávia S. B. Dias, Wanderson Roberto da Silva, Mônica da Silva-Nunes, Alanderson Alves Ramalho

**Affiliations:** 1 Graduate Program in Public Health, Federal University of Acre, Rio Branco 69920-900, AC, Brazil; alanderson.ramalho@ufac.br; 2 Graduate Program in Food, Nutrition and Food Engineering, São Paulo State University (UNESP), Araraquara 14800-903, SP, Brazil; wanderson.silva@unesp.br; 3 Department of Medicine, Center for Biological and Health Sciences, Federal University of São Carlos, São Carlos 13565-905, SP, Brazil; monicasn@ufscar.br

**Keywords:** validity, reliability, nutritional surveys, students, food choice, motivation

## Abstract

This study aimed to test the factorial structure of the Eating Motivation Survey (TEMS) using Confirmatory Factor Analysis (CFA) in a sample of 632 university students from the Western Brazilian Amazon. A cross-sectional study was conducted between December 2022 and April 2023 with participants of both sexes, aged 18 or older. In addition to CFA, psychometric analyses were performed, and a Structural Equation Model was developed to examine the relationships between individual characteristics (age, sex, and Body Mass Index (BMI)) and the TEMS constructs. The results showed that 58.3% of participants were female, with a mean age of 25.29 years. The CFA supported an eight-factor model (health, natural concerns, socialization, price, visual appeal, weight control, emotional control, and social image) with 24 items, presenting good validity and reliability indices. Older individuals and those with lower BMIs prioritized health, natural concerns, and weight control, while younger participants, women, and those with higher BMIs were more influenced by emotional control. The findings contribute to understanding eating motivations in culturally diverse contexts and may support strategies aimed at promoting healthier dietary behaviors and preventing diet-related chronic diseases.

## 1. Introduction

Food choice for consumption can be defined as a concurrent and reinforced interaction influenced by a variety of factors. Eating habits and the complex process of food selection are driven by an interplay of physiological, genetic, epigenetic, economic, social, and behavioral factors, as well as sensory characteristics of foods, and the activities of the food industry aimed at promoting them [1,2].

Food is so fundamental and is woven into human life in multiple dimensions, engaging nearly all routine activities, including leisure, arts, sex, and work. Nutrition, and particularly food choice, is as central to biological evolution as any other activity [3].

Food choices develop throughout an individual’s life trajectory, shaped by predecessors and by individual and historical temporal contexts, and are susceptible to changes related to the transitions experienced at different life stages. Each new food experience contributes to shaping subsequent choices, allowing for the assessment of the impact of economic trends and the food system on dietary decisions [4].

When made by the general population, these choices are generally guided by factors that encompass both broader living conditions—such as income level, local urbanization, and access to food variety—as well as individualized characteristics, including educational level, age group, and family food culture [5]. Glanz et al. [6] pointed out that food environments exert a strong influence on dietary choices, based on demographic, psychosocial, and perceived environmental variables. Among these environments—such as schools, workplaces, universities, and churches—there is an especially important role in promoting healthy eating practices.

Studies conducted with university students have extensively explored dietary habits, physical activity levels, body composition, and other related aspects. However, there remains a gap in the literature regarding research addressing the influence of motivations on food choices within the university environment [7]. This aligns with the findings of Maciel et al. [5], who evaluated the nutritional status of a sample of 303 university students from the interior of São Paulo, reporting a prevalence of overweight of 16.8% among women and 47.7% among men. The authors also noted that the food environment was not assessed, highlighting the need for further studies that examine causal relationships between determinants of these variations in academic communities.

To obtain information on food choices, instruments such as the Eating Motivation Survey (TEMS), among others, have been developed to capture in-depth the variety of these motivations. Other tools have also been developed to assess motivations for food choice, such as the Food Choice Questionnaire (FCQ) and the more recent EATMOT scale, which explores psychological, emotional, and social attitudes toward eating [8]. However, TEMS remains one of the most comprehensive instruments for identifying behavioral and contextual determinants of food choice.

Understanding trends in motivations, values, and challenges derived from research using instruments that measure food choices can contribute to improving educational practices, interventions, and nutritional recommendations in public health for individuals, groups, communities, and populations [9]. Through its cross-cultural adaptation and validation for Brazil, Moraes and Alvarenga [10] concluded that the Brazilian version of the reduced TEMS (15 factors—45 items) is adequate, representing a comprehensive and useful instrument.

The self-administered instrument format, which does not require respondents to specify exact foods consumed, encourages individuals to reflect on their overall daily eating behavior and subsequently determine the motivations they consider “appropriate” for each eating occasion [11,12]. Psychometric instruments are essential for studies involving human behavior, as they allow for the collection of valid and reliable data on conduct and cognitive processes. Accordingly, TEMS has demonstrated utility across different cultures and is presented as an instrument applicable in diverse contexts [13].

Considering that this current scale had not previously been applied to the university student context, the aim of this study is to test the factorial structure of the Eating Motivation Survey using Confirmatory Factor Analysis (CFA) and to apply Structural Equation Modeling (SEM) in order to assess the model fit and conduct a psychometric analysis of the instrument in a sample of university students from the Western Brazilian Amazon.

## 2. Materials and Methods

### 2.1. Sample Characteristics

This was a non-probabilistic cross-sectional study with university students. Initially, participants were recruited through social media, in-class announcements, and email invitations sent to students’ institutional addresses via Academic coordination, explaining the study’s purpose and characteristics. No payment or incentives were offered for participation. Inclusion criteria were: (i) being a regularly enrolled undergraduate student at the Federal University of Acre, main campus; and (ii) having internet access. Exclusion criteria included students under 18 years old and pregnant women.

Participants in this study comprised all undergraduate students from courses at the Rio Branco, Acre State, Brazil, Campus of the Federal University of Acre, of both sexes, over 18 years old, who answered the survey between December 2022 and April 2023.

### 2.2. Sample Size Calculation

Several recommendations exist for determining sample size in psychometric studies [14,15,16], and these are not always consistent. However, a commonly used rule of thumb in the literature for conducting Confirmatory Factor Analysis (CFA) is to adopt 10 respondents per item of the instrument under investigation [16]. For the TEMS, the minimum sample size was calculated based on a 10:1 ratio for the 45 items, accounting for a 20% dropout rate, which is common in cross-sectional studies. Thus, the minimum required sample for CFA was 563 individuals.

### 2.3. The Eating Motivation Survey (TEMS)

The TEMS was developed by Renner et al. [17] in a German context to investigate motives behind food choices that trigger eating behavior in daily life. The original scale comprises 45 items grouped into 15 factors: Preference (items 2, 3, 23); Habits (items 10, 40, 45); Need and Hunger (items 1, 18, 36); Health (items 8, 17, 44); Convenience (items 4, 21, 33); Pleasure (items 16, 22, 39); Traditional Eating (items 11, 14, 43); Natural Concerns (items 20, 26, 29); Socialization (items 15, 27, 34); Price (items 7, 28, 41); Visual Appeal (items 24, 30, 35); Weight Control (items 6, 13, 38); Emotional Control (items 5, 12, 37); Social Norms (items 19, 25, 32); Social Image (items 9, 31, 42). In the original study, TEMS response options were arranged on a seven-point Likert scale ranging from “never” (1) to “always” (7). However, Moraes and Alvarenga [10], who conducted the cross-cultural adaptation of TEMS for Brazilian Portuguese, recommended a five-point Likert scale ranging from “never” (1) to “always” (5). The Portuguese version of TEMS with this response format was applied in the present study.

### 2.4. Psychometric Analysis

Given that the Eating Motivation Survey (TEMS) is grounded in an a priori theoretical measurement model, a confirmatory psychometric approach was adopted. Confirmatory Factor Analysis (CFA) was conducted to evaluate the factorial structure of the instrument, followed by the assessment of convergent and discriminant validity, measurement invariance of the refined model, and reliability. All analyses were performed using R software (version 4.1.2) in the RStudio environment (version 2023.06.1), employing the lavaan, semTools, and psych packages.

#### 2.4.1. Psychometric Sensitivity

Psychometric sensitivity was assessed through a descriptive analysis of responses for each item on each scale, including mean, median, mode, standard deviation, skewness, kurtosis, standard error, and the percentage distribution of participants across response categories (see Supplementary Material, Tables S1 and S2). When absolute values were below 3 for skewness and below 7 for kurtosis, psychometric sensitivity was confirmed, suggesting no severe violation of the assumption of data normality [14,16].

#### 2.4.2. Factorial Validity

Confirmatory Factor Analysis (CFA) was conducted using the robust Weighted Least Squares Mean and Variance Adjusted (WLSMV) estimation method [14,16]. Model fit for each scale was evaluated using a set of indices, namely: chi-square divided by degrees of freedom (χ^2^/df), Comparative Fit Index (CFI), Tucker–Lewis Index (TLI), Standardized Root Mean Square Residual (SRMR), and Root Mean Square Error of Approximation (RMSEA) with a 90% confidence interval (CI90%). According to the specialized literature [16,18], χ^2^/df ≤ 2, CFI and TLI ≥ 0.95, SRMR ≤ 0.08, and RMSEA < 0.08 indicate adequate model fit, supporting factorial validity. Acceptable but less optimal fit is defined as χ^2^/df < 5, CFI and TLI ≥ 0.90, SRMR ≤ 0.09, and RMSEA < 0.10. As χ^2^/df tends to inflate with large sample sizes, it was not considered in isolation. Factor loadings (λ) of scale items are also critical parameters, with values above 0.30 generally accepted; however, values above 0.40 are expected for optimal model fit [14,16].

For measurement model refinement aimed at improving fit, Modification Indices (MI), calculated from Lagrange Multipliers, were inspected. Values above 11 suggest a need to revise the measurement model, either by allowing covariance between item errors or by removing items highly correlated with other items and distinct factors [16].

During the model refinement process, both theoretical and statistical criteria were considered for item and factor retention. Items presenting low factor loadings were excluded, and factors with weak theoretical support or redundancy across constructs were also removed. These procedures followed the recommendations of Hair et al. [14], Kline [15], and Marôco [16] for psychometric robustness.

#### 2.4.3. Convergent and Discriminant Validity

To investigate whether the set of items proposed for each scale factor adequately represents it, the average variance extracted (AVE) was calculated based on factor loadings and item errors [19,20]. Convergent validity of a factor is established when the AVE value is ≥0.50 [14,16]. Discriminant validity was examined using the Fornell–Larcker criterion by comparing the AVE of each latent factor with the squared correlations between factors, in order to verify whether each construct shared more variance with its indicators than with other constructs [20].

#### 2.4.4. Factorial Invariance

After determining the measurement model adjusted for the scale under investigation in the study sample, invariance tests were conducted to verify the equivalence of the model across different groups, allowing for future comparisons. First, the total sample was randomly split into two equal subsamples, which were submitted to invariance testing to assess external validity, i.e., to investigate the model’s applicability in independent observations. Subsequently, invariance testing was performed comparing groups by sex (male vs. female), age (≤24 years vs. ≥25 years), and BMI (≤25.9 kg/m^2^ vs. ≥26.0 kg/m^2^). The age and BMI group divisions were based on the sample mean. Multi-group confirmatory factor analysis was conducted for the invariance tests, and model fit indices (CFI and RMSEA) obtained from the configural (baseline—equivalence of factorial structure without equality constraints), metric (equivalence of factor loadings), scalar (equivalence of factor loadings and thresholds), and strict (equivalence of factor loadings, thresholds, and residuals) models were compared using the differences (Δ) in reduction (e.g., CFI values: metric—configural). When ΔCFI values were below 0.01, supplemented by ΔRMSEA values below 0.015, model invariance was confirmed, indicating strong equivalence across groups [21,22].

#### 2.4.5. Reliability

To investigate whether the set of items proposed for each factor was consistent and reliably measured the underlying construct, composite reliability (CR) [13,18,19], ordinal alpha coefficient (α) [23], and omega coefficient (ω) [24] were computed. A value ≥ 0.60 is considered acceptable for detecting internal consistency. However, a value ≥ 0.70 in at least one of the indices (preferably in all of them) is recommended to confirm reliability [14].

### 2.5. Structural Equation Modeling

To examine the relationship between participants’ individual characteristics (sex, age, and BMI) and the scale constructs (considering the measurement model adjusted for the current study sample), a structural equation model was constructed. Independent variables included sex [female (0), male (1)], age [≤24 years (0), ≥25 years (1)], and BMI [≤25.9 kg/m^2^ (0), ≥26.0 kg/m^2^ (1)], while the dependent variables were the scale factors. Hypothetical causal paths (β) representing the relationships between variables were estimated and compared against critical z-ratios, considering a significance level of 5%. Only significant β values were retained in the final (i.e., refined) structural model. Model fit was evaluated using the CFI, TLI, and RMSEA indices, with adequacy thresholds as described previously in the Factorial Validity subsection [16].

### 2.6. Ethical Aspects

Data was collected via an electronic questionnaire using Google Forms^®^. This study was conducted with the approval of the Research Ethics Committee of the Federal University of Acre, under Report number 5,740,597, in the Brazilian Certificate of Submission for Ethical Assessment (CAAE: 63466722.0.0000.5010), and participants provided written informed consent prior to participation.

## 3. Results

Of the 655 undergraduates who responded to the questionnaire, 23 were excluded for not answering all TEMS items, resulting in a final sample of 632 students. Among these, 58.3% were female, 72.8% were aged between 20 and 29 years, with a mean age of 25.29 years (standard deviation = 7.39).

Table 1 presents the set of psychometric properties (obtained via CFA) used to evaluate the TEMS model in the study sample.

Considering that the psychometric properties of the original TEMS measurement model were unsatisfactory for the current study sample, modifications were undertaken to obtain an adjusted structure (Table 1). The first modification involved the removal of item 1, which had a factor loading below 0.30 (Table 2); however, this did not alter model fit. Before proceeding with further exclusions, Modification Indices (MI) were examined, revealing extreme values (>60) indicating covariance between item errors within the same factor (MI: item4/item33 = 176.18; item21/item33 = 81.42; item5/item12 = 61.53) and between distinct factors (MI: item45/item43 = 135.25; item36/item32 = 107.80; item14/item15 = 87.83; item23/item22 = 70.43; item36/item8 = 67.31), suggesting similar item content.

After allowing only covariances between errors of items within the same factor, the model continued to show unsatisfactory fit (χ^2^/df = 3.32; CFI = 0.88; TLI = 0.86; SRMR = 0.07; RMSEA = 0.06, 90% CI = 0.058–0.061), prompting further strategies.

Due to compromised convergent validity and/or reliability of five factors (Table 3: Habits; Need and Hunger; Convenience; Traditional Eating; Social Norms), the item with the lowest loading from each factor was removed (leaving only two items per factor). This modification resulted in acceptable model fit (χ^2^/df = 3.02; CFI = 0.91; TLI = 0.90; SRMR = 0.06; RMSEA = 0.06, 90% CI = 0.054–0.060), but AVE values and reliability indices remained unsatisfactory. Consequently, these factors were removed from the measurement model, resulting in improved fit (χ^2^/df = 3.18; CFI = 0.94; TLI = 0.93; SRMR = 0.06; RMSEA = 0.06, 90% CI = 0.055–0.063), yet still below the desired thresholds.

The Preference and Pleasure factors continued to show a lack of convergent validity. To address this, the item with the lowest factor loading was removed from each factor; however, these changes worsened reliability indices. Therefore, these factors were also eliminated.

**Figure 1:**
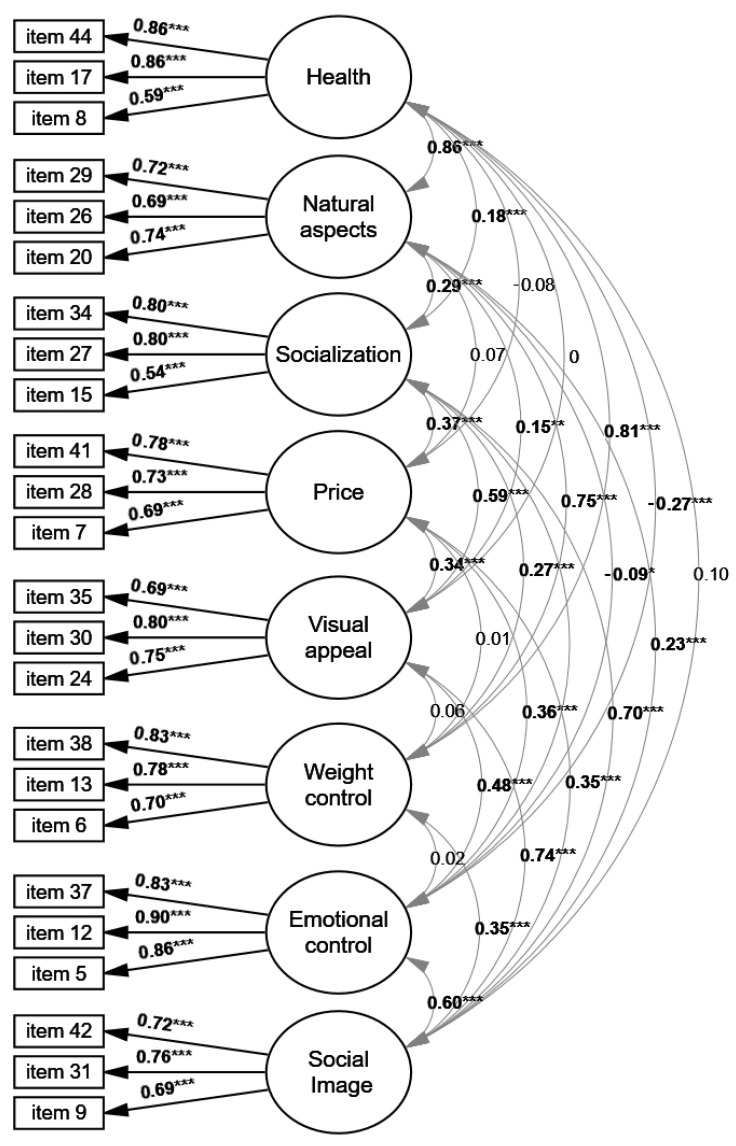
Refined and fitted measurement model showing the grouping of items, their respective factor loadings, and the latent factors they represent in the Eating Motivation Survey (TEMS) for the sample of the present study. Note: Black arrows indicate the factor loadings of the items (all statistically significant), represented by rectangles. Ellipses represent latent variables. Double-headed arrows between latent variables indicate the correlations among them, with significant correlations shown in bold. * *p* < 0.05; ** *p* < 0.01; *** *p* < 0.001.

The exclusion of seven TEMS factors from the original model, adapted and translated for Brazil, resulted in a refined and adjusted model comprising 24 items distributed across 8 factors (Figure 1). This model demonstrated adequate factorial validity with factor loadings above 0.54 (Table 2), good convergent validity and reliability indices (Table 3), and most of the correlations between factors were significant (Supplementary Table S3). Discriminant validity was adequate for most of the retained factors. However, partial violations of the Fornell–Larcker criterion were observed for the factors Health and Natural Concerns (AVE < r^2^), indicating a substantial conceptual overlap between these constructs in the present sample.

Regarding the Social Image factor, a borderline omega value (ω = 0.68) was observed; however, the remaining reliability indices were adequate, which does not compromise the overall reliability of the construct.

The refined and adjusted measurement model of the TEMS for the current study sample was applied to factorial invariance tests across different groups, with results presented in Table 4. There was invariance across all groups analyzed (subsamples; sex; age; BMI), indicating equivalence of the measure.

Regarding the structural model tested (Table 5) with TEMS factors (i.e., including all dependent and independent variables), some relationships were found to be non-significant and were therefore removed, resulting in a refined structural model. In this model, older participants were more likely to choose foods for health reasons (β = 0.20), natural concerns (β = 0.22), and weight control (β = 0.22), whereas younger participants chose food for emotional control (β = −0.14).

Individuals with lower BMI selected food emphasizing health (β = −0.19), while those with higher BMI chose food for emotional control (β = 0.30). Additionally, compared to men, women selected food for socialization purposes (β = −0.11) and emotional control (β = 0.24).

## 4. Discussion

The present study conducted a confirmatory factor analysis (CFA) to examine the structure of the TEMS among the university population. Fit indices indicated that the 8-factor, 24-item model most closely approached the quality levels usually required [15]. The CFA results provided additional evidence supporting factorial and convergent validity, as well as good reliability indices and significant correlations between factors. It was observed that older participants chose food more for “Health,” “Natural Concerns,” and “Weight Control,” whereas younger participants chose food for “Emotion Regulation.” Individuals with lower BMI selected food prioritizing “Health,” while those with higher BMI chose food for “Emotion Regulation,” which may highlight the need for multidisciplinary care in overweight and obesity management. Additionally, compared to men, women were more likely to choose food for “Socialization” and “Emotion Regulation.”

Several international studies [25,26,27,28], including one with a university population [29], and some national studies [2,30,31,32,33,34,35] have employed the TEMS in their target populations. However, the factorial structure of the original short scale (45 items and 15 factors) was maintained, and no CFA was conducted in these samples.

Brazilian studies [36,37] tested the psychometric properties of TEMS, with the latter comparing it to the Food Choice Questionnaire (FCQ), which served as the basis for TEMS development. Findings indicated that the 45-item, 15-factor structure showed “acceptable” fit but had limitations in reliability (assessed by Cronbach’s alpha, with some values below 0.70 and others borderline values) and in inter-factor correlations (some were not significant). These results suggest that the 45-item, 15-factor model may be fragile and that psychometric investigations in different Brazilian contexts are necessary to ensure adequate capture of food choice motives using TEMS.

Although the present study did not aim to compare the TEMS with other instruments, it is relevant to note that the Food Choice Questionnaire (FCQ) and the EATMOT scale address similar motivational constructs from distinct theoretical perspectives. The FCQ emphasizes the cognitive and attitudinal determinants of food choice, while the EATMOT explores the emotional, social, environmental, and ethical dimensions of eating behavior, focusing on attitudes related to health, pleasure, convenience, sustainability, and moral values [8]. The TEMS, in turn, offers a multidimensional behavioral model that integrates these motivational aspects into a single structure [36], which justifies its use as the central instrument in this study.

An evaluation was performed against the Brazilian version of the scale, adapted and validated by Moraes and Alvarenga [10], as well as the validation conducted for the Mexican population [12]. In Brazil, the scale preserved the original structure, showing satisfactory fit indices. In Mexico, the final version resulted in 11 factors and 34 items, a higher number than obtained in the present study, but with adequate fit indices and internal consistency ranging from 0.74 to 0.86, values that are similar to those observed in our analyses.

Another validation study conducted in Turkey indicated the retention of the 15 original factors, showing “acceptable fit” similar to that obtained in the present study, with CFI = 0.958 (>0.97), and “good fit” for TLI = 0.963. Compared with our findings, the Turkish study reported slightly lower values, but still indicated “good fit” with RMSEA = 0.037 (<0.05), which was considered >0.90, confirming “good fit,” and RMR = 0.027 (<0.05). These results demonstrate that the validity and reliability of the abbreviated TEMS version are sufficient, according to construct validity for the Turkish population [36]. A Finnish study in an adult population (n = 1048) also retained the 15 original factors, yielding results similar to ours, with CFI = 0.929 and RMSEA = 0.042, indicating excellent fit and confirming the global validity of TEMS in the Finnish sample [38].

The factorial invariance analysis demonstrated that the TEMS model exhibited equivalence across the different groups evaluated, allowing valid comparisons. The results indicated that the 24-item, 8-factor model was invariant across independent subsamples, sexes, age groups, and BMI categories of the participants.

This supports the external validity of the TEMS measurement model and the plausibility of comparing groups to analyze food choice motive scores for daily life without interference from the scale’s operationalization. Overall, discriminant validity was adequate for most factors; however, a partial conceptual overlap between health and natural concerns was observed, reflecting the theoretical proximity between these constructs.

Sproesser et al. [39] investigated the consistency and measurement invariance of the fifteen basic motives included in TEMS among countries with highly diverse food environment such as the USA, India, and Germany. The study aimed to examine whether eating behavior in German samples also underlies eating behavior in the USA and India, revealing that the scale structure is generalizable across these countries. Furthermore, measurement invariance of TEMS was investigated among the three countries, and despite the complexity of the fifteen-factor model, fit indices indicated reasonable model fit (for the total sample: χ^2^/df = 4.03; standardized root mean square residual [SRMR] = 0.063; root mean square error of approximation [RMSEA] = 0.064, 95% CI 0.062–0.066). Only the comparative fit index (CFI) has fallen below the recommended threshold (for the total sample: CFI = 0.84). Overall, 181 of 184 item loadings exceeded the recommended threshold of 0.30.

Moreover, the TEMS factorial structure was invariant across countries regarding factorial configuration and factor loadings (configural vs. metric invariance model: ΔCFI = 0.009; ΔRMSEA = 0.001; ΔSRMR = 0.001). It is worth noting some differences compared to the Amazonian context, as the application of the scale in the above countries used the original German structure with one additional item compared to the Brazilian instrument by Moraes et al. [40], and a larger Likert response scale (1–7). Psychometric analysis across the three populations yielded less robust results compared to our evaluations. Additionally, forty-three of the forty-six items from the original German scale had invariant intercepts across these countries. Thus, food choice motives were remarkably consistent in structure despite marked differences in food environments.

Another important consideration highlighted by Sproesser et al. [39] is that the investigation of measurement invariance showed that latent means for fourteen of the fifteen food choice factors can be compared across countries in future studies with representative samples, consistent with the findings of the present study.

In a study conducted with people living with HIV (PLWHIV) in Botucatu (SP), some observations were made regarding the TEMS scale analysis. The author noted that although the overall Cronbach’s α for the scale (0.79) was considered satisfactory (0.7–0.9), individual dimensions showed low values and internal consistency below expectations, indicating response variability. The study findings suggested that this instrument may not be suitable for assessing food choices among PLWHA [41].

Based on the confirmatory factor analysis (CFA) applied in the present study, the structure of the original instrument was modified to achieve better model fit, with the exclusion of twenty-one items and a reduction from fifteen to eight factors. Such modifications are common and hypothesized across different populations and may be explained by cultural differences, including regional variations, food access, socioeconomic factors, and dietary culture. Although the studies by Moraes et al. [35] and Sproesser et al. [36] were conducted in Brazil, our results differed in terms of the resulting TEMS structure; these differences can be attributed to the regional contexts of the studies and the heterogeneity of the country.

The reduction in the number of items and factors in the refined model should be interpreted as a theoretically and empirically justified adaptation, and not as a loss of content, since the exclusion of factors such as “Convenience,” “Habits,” and “Social Norms” reflected their poor psychometric performance in this specific cultural and university context, where these motives are likely less relevant. Similar refinements of the model have been observed in other cross-cultural validations of the TEMS [13,37], corroborating the idea that motivational domains may vary across populations. Therefore, the final eight-factor model preserves the theoretical coherence of the original instrument. Furthermore, this pattern of factor exclusion is theoretically consistent with the eating behavior characteristics of the studied group, in which emotional and social aspects exert a greater influence on food choices than convenience or routine. This suggests that the reduced model reflects the most relevant motivational structure.

It is important to note that this study focused exclusively on evaluating the internal structure and construct validity of the reduced TEMS version. External validation through correlations with dietary assessments (such as food frequency questionnaires, dietary diaries, or ecological momentary assessments) was not performed. Future investigations by the broader research community could examine these associations to verify whether the motivational factors identified by TEMS predict actual eating behaviors. Such work would contribute to expanding the evidence base for the criterion of the instrument.

Examining the structural model, one of the most prominent motives was “Emotional Control,” a factor highlighted by Freitas [42] in a qualitative study, where negative emotions significantly influence food motivation and desire, leading to food intake as a strategy to mitigate emotional intensity. This finding was also corroborated by Penaforte et al. [43] in a study with university students, which indicated that emotional eating is directly related to an individual’s mood and stress. Such phenomena can be explained by understanding that eating behavior (what, how much, and when) is driven by complex neural processes, combined with dietary culture, exposure, variety, availability, prior individual experiences, personal factors such as sex, income, and age, as well as digestive consequences, among others, all of which directly influence specific food choices. These motivations arise primarily from a combination of physiological and psychological needs [44,45].

The structural model further indicated that individuals with higher BMI are motivated to eat for emotional control, whereas those with lower BMI choose foods based on health-related motives, as was also affirmed by Rodrigues et al. [46] in their cross-sectional study with 675 consumers from a restaurant in Florianópolis (SC). In the study, being overweight was positively associated with not choosing rice and beans, a preference for less varied salads, and larger portion sizes when BMI was adjusted for sex, age, education, marital status, and other food choice variables. Compared with the TEMS validation findings in Brazil, Moraes [40] reported divergent results, where individuals with BMI ≥25 kg/m^2^ were more likely to choose foods for convenience and weight control and less for tradition and habits. Renner et al. [17], the developers of TEMS in Germany, found that individuals with lower BMI tend to choose food more based on preference, need and hunger, and health, whereas those with BMI ≥ 25 kg/m^2^ reported choosing food more for weight control, emotion regulation, and social norms, showing factors similar to those found in the refined model of the present study.

In the Brazilian study by Sproesser et al. [36], individuals with higher BMI showed significantly higher scale means for the motives “Convenience,” “Pleasure,” “Price,” “Visual Attraction,” “Weight Control,” “Emotion Regulation,” “Social Norms,” and “Social Image.” In contrast, participants with lower BMI had significantly higher scale means for the motives “Need and Hunger” and “Health.” Motives such as “Emotion Regulation” and “Health” also emerged in higher and lower BMI groups, respectively, which is similar to the findings of this study.

Boek et al. [47], evaluating Californian university students and their food choices, described sex as a significant factor in dietary decisions. Men were more motivated by price and taste. Moraes [40] reaffirmed these results, reporting that among young women, health was the most prominent factor, followed by convenience, pleasure, price, and weight control. These characteristics differ from those observed in the present study, where women, compared to men, chose food for socialization and emotion regulation.

Food motivations are complex and involve an interaction of factors that determine the type of food chosen [48]. Health and nutrition concerns are essential considerations in any food choice; however, they may conflict with concerns about price, availability, and taste, further compromising the decision to choose healthy food [49].

There is a pressing need for nutritional education and psychological support in university populations, considering their lifestyle and eating patterns, as they are subject to constant changes due to the demands placed upon them, particularly as academic careers and age progress. According to Lourenço [50] and Canesqui and Garcia [51], modern life imposes changes related to time, work, convenience, productivity demands, cost, and diversity influenced by media and globalization. These factors demonstrate that eating behavior cannot be explained solely from the perspective of eating practices—which involve choice, quantity, and preparation—but must consider the broader context of an individual’s daily life dynamics.

Freitas (2020) [42] highlights that understanding and guiding young people based on dietary difficulties can help mitigate challenges in coping with the demands of adult life. Accordingly, recognizing emotional factors and other socioeconomic and dietary behavior motivators is fundamental for identifying strategic approaches to support individuals struggling to adopt healthy eating practices. In the university environment, promoting awareness about the importance of proper nutrition is essential, as healthy lifestyle habits contribute to overall health, quality of life, and the improvement of dietary habits and anthropometric status [7].

It should be emphasized that this study was conducted among university students from the Western Brazilian Amazon, a region where eating motivations—shaped by factors such as limited access, food insecurity, socioeconomic constraints, and food availability—may differ substantially from other regions of the country.

### Limitations and Strengths of the Study

Regarding the limitations of this study, data were collected online, and the use of a convenience sample may have introduced selection bias, as participation depended on internet access and willingness to complete online questionnaires. This may have resulted in the overrepresentation of individuals with a greater interest in nutrition-related topics. In addition, the online format restricted the length of the data collection instrument, and some potentially relevant variables—such as lifestyle behaviors and physical activity practices—were not included. Despite these limitations, the invitation to participate was extended to all students at the institution, encompassing undergraduates from all academic programs, and the large number of respondents strengthens the internal consistency and robustness of the findings. Although the results cannot be generalized to other university populations, the broad institutional coverage enhances the relevance of the observed patterns.

Furthermore, data collection took place during a transitional period following the COVID-19 pandemic, a context in which digital communication and online academic engagement remained highly prevalent among university students. This likely mitigated access barriers and reduced the magnitude of potential selection bias. Nevertheless, this limitation may be addressed in future studies through the adoption of probabilistic or stratified sampling strategies, as well as by combining online and in-person data collection methods. Notwithstanding these considerations, it is unlikely that the potential selection bias substantially compromised the internal validity of the study, as the primary objective was to examine the psychometric properties and internal factor structure of eating motivation constructs within a defined sample, rather than to estimate population prevalences or parameters. Moreover, the observed patterns were consistent with theoretical expectations and with findings previously reported in the literature.

Other limitations may be associated with information bias, since self-reported weight and height can generate inaccuracies in BMI measurement. However, other surveys also rely on self-reported anthropometry, particularly Surveillance of Risk and Protective Factors for Chronic Diseases by Telephone Survey (in Brazil—VIGITEL), which has been conducted annually in Brazil for over 15 years. Moreover, studies have demonstrated the validity of using self-reported data compared with data from in-person surveys [52,53]; thus, obtaining self-reported weight and height is widely used and recommended in health research [54,55,56], including in Rio Branco [53].

Another limitation is the absence of cross-validation analysis, which would allow for the assessment of the stability of the factor model in independent samples. Future studies are encouraged to conduct such analyses to verify the stability and replicability of the structure identified in this study. Similarly, the internal consistency, factor adequacy, convergent validity, and discriminant validity of the reduced version of the TEMS were examined in the present study. Other complementary approaches to validity and reliability assessment were not applied. Particularly, temporal stability was not assessed, as test–retest reliability could not be conducted due to practical constraints related to data collection and the characteristics of the study population. In addition, convergent and discriminant validity were evaluated only within the internal structure of the instrument, and external validation through comparisons with other theoretically related and previously validated measures was not performed. Future studies are encouraged to incorporate additional strategies, such as test–retest reliability and more stringent assessments of discriminant validity, including the Heterotrait–Monotrait ratio (HTMT), as well as external validation procedures, preferably in the same target population, to further confirm the distinctiveness and temporal stability of the constructs.

As strengths of this research, it is important to note that, despite the use of a convenience sample, the study included a substantial number of participants. Even after excluding cases with incomplete data for confirmatory factor analysis, the final sample size was sufficient to meet the study objectives. The refined eight-factor structure demonstrated adequate model fit according to multiple fit indices, and convergent validity was supported by adequate factor loadings and satisfactory average variance extracted values, indicating that the items of each factor consistently represented their underlying constructs.

Considering that the instrument is relatively new for use in Brazilian research, this work is pioneering both in the academic context and in the proposed analysis for comparison with the Brazilian validation study. Although the final structural model indicates an instrument reduced by almost half in terms of factors and items compared to the original model, we believe that it remains a valid and reliable tool for the sample of university students from Rio Branco. Therefore, the results obtained reinforce the importance of a better understanding of the dietary choices of the population in a country like Brazil, particularly in the Northern region, being suitable for future reference in studies involving comparable populations.

## 5. Conclusions

This study establishes a valid and reliable model of the Eating Motivation Survey (TEMS) for university students in Rio Branco (Brazil), revealing how individual characteristics (such as age, BMI, and sex) were associated with food choice motives. These findings advance the understanding of eating motivations within a sociocultural and behavioral framework and may inform strategies to promote healthier food choices and prevent diet-related chronic diseases. Nevertheless, further studies are necessary to confirm the external validity of the reduced TEMS version through cross-validation analyses and comparisons with dietary assessments or observed eating behaviors. Such investigations will help assess whether the motivational factors identified by the scale are associated with actual dietary patterns, thereby strengthening its criterion validity and supporting its potential application in future nutrition and public health research.

## Figures and Tables

**Table 1:** Psychometric properties of the Eating Motivation Survey (TEMS) when tested with the sample of the present study.

	Original Model (45 Items and 15 Factors)	Refined and Adjusted Model (24 Items and 8 Factors)
χ^2^	2946.72	679.95
df	840	224
CFI	0.87	0.96
TLI	0.84	0.95
RMSEA [IC90%]	0.06 [0.06–0.07]	0.06 [0.05–0.06]
SRMR	0.07	0.05
λ	0.20–0.90	0.54–0.90
AVE	0.19–0.75	0.51–0.75
CR	0.38–0.90	0.76–0.90
α	0.37–0.88	0.71–0.88
ω	0.36–0.87	0.68–0.87

Source: Prepared by the authors, 2023. Note: χ^2^: chi-square test, df: degrees of freedom, CFI: Comparative Fit Index, TLI: Tucker–Lewis Index, RMSEA: Root Mean Square Error of Approximation, 90%CI: 90% confidence interval, SRMR: Standardized Root Mean Square Residual, λ: item factor loading (minimum and maximum values), AVE: average variance extracted (minimum and maximum values), CR: composite reliability (minimum and maximum values), α: ordinal alpha (minimum and maximum values), ω: omega (minimum and maximum values).

**Table 2:** Factor loadings of the items from the Eating Motivation Survey (TEMS) considering the original measurement model and the refined and adjusted model for the sample of the present study.

Item	Original Model (45 Items and 15 Factors)	Refined and Adjusted Model (24 Items and 8 Factors)
1	0.20 *	-
2	0.70	-
3	0.60	-
4	0.60	-
5	0.85	0.86
6	0.69	0.70
7	0.64	0.69
8	0.65	0.59
9	0.68	0.69
10	0.45	-
11	0.49	-
12	0.90	0.90
13	0.77	0.78
14	0.45	-
15	0.60	0.54
16	0.60	-
17	0.85	0.86
18	0.45	-
19	0.66	-
20	0.73	0.74
21	0.64	-
22	0.74	-
23	0.75	-
24	0.78	0.75
25	0.72	-
26	0.70	0.69
27	0.78	0.80
28	0.78	0.73
29	0.72	0.72
30	0.77	0.80
31	0.75	0.76
32	0.44	-
33	0.71	-
34	0.78	0.80
35	0.69	0.69
36	0.57	-
37	0.84	0.83
38	0.84	0.83
39	0.72	-
40	0.59	-
41	0.78	0.78
42	0.73	0.72
43	0.38 *	-
44	0.85	0.86
45	0.65	-

* Factor loading less than 0.40. A hyphen indicates that the item is not part of the model.

**Table 3:** Convergent validity and reliability properties of the factors of the Eating Motivation Survey (TEMS) considering the original measurement model and the refined and adjusted model for the sample of the present study.

	Original Model (45 Items and 15 Factors)				Refined and Adjusted Model (24 Items and 8 Factors)			
Factor	AVE	CR	α	ω	AVE	CR	α	ω
Preference	0.47 *	0.72	0.72	0.67 ^‡^	-	-	-	-
Habits	0.32 *	0.58 ^†^	0.54 ^†^	0.52 ^†^	-	-	-	-
Need and Hunger	0.19 *	0.38 ^†^	0.37 ^†^	0.36 ^†^	-	-	-	-
Health	0.62	0.83	0.81	0.80	0.61	0.82	0.81	0.80
Convenience	0.43 *	0.69 ^†^	0.58 ^†^	0.66 ^†^	-	-	-	-
Pleasure	0.48 *	0.73	0.73	0.70	-	-	-	-
Traditional Food	0.20 *	0.42 ^†^	0.40 ^†^	0.39 ^†^	-	-	-	-
Natural Concerns	0.51	0.76	0.76	0.72	0.51	0.76	0.76	0.72
Socialization	0.53	0.77	0.71	0.73	0.52	0.76	0.71	0.73
Price	0.54	0.78	0.77	0.74	0.54	0.78	0.77	0.74
Visual Appeal	0.56	0.79	0.78	0.75	0.56	0.79	0.78	0.75
Weight Control	0.59	0.81	0.81	0.78	0.59	0.81	0.81	0.78
Emotional Control	0.75	0.90	0.88	0.87	0.75	0.90	0.88	0.87
Social Norms	0.38 *	0.64 ^†^	0.57 ^†^	0.58 ^†^	-	-	-	-
Social Image	0.52	0.76	0.76	0.68 ^‡^	0.52	0.76	0.76	0.68 ^‡^

Source: Prepared by the authors, 2023. Note: AVE: average variance extracted, CR: composite reliability, α: ordinal alpha, ω: omega. * AVE value less than 0.50 suggesting a lack of convergent validity. † Value less than 0.70 in all reliability indices, violating this property. ‡ Value less than 0.70 or borderline in one of the reliability indices, not violating the overall reliability of the factor.

**Table 4:** Invariance tests of the refined and adjusted measurement model of the Eating Motivation Survey (TEMS) for the sample of the present study.

	Group							
	Subsamples		Sex		Age		BMI	
	(Subsample1: n = 316 vs. Subsample2: n = 316)		(Men: n = 264 vs. Women: n = 360)		(≤24 Years: n = 400 vs. ≥25 Years: n = 224)		(≤25.9 kg/m^2^: n = 397 vs. ≥26.0 kg/m^2^: n = 227)	
Model Comparison	ΔCFI	ΔRMSEA	ΔCFI	ΔRMSEA	ΔCFI	ΔRMSEA	ΔCFI	ΔRMSEA
Metric—Configural (factor loadings)	0.001	0	−0.002	0.003	−0.001	0	−0.001	0.002
Scalar—Metric (thresholds)	0.002	−0.006	0.001	−0.005	0.001	−0.004	0.001	−0.005
Strict—Scalar (residuals)	0	0	0	0	0	0	0	0

Source: Prepared by the authors, 2023. Note: BMI: Body Mass Index, CFI: Comparative Fit Index, RMSEA: Root Mean Square Error of Approximation, Δ: Difference between comparison models.

**Table 5:** Structural model tested to analyze the relationships between the Eating Motivation Survey (TEMS) factors and participants’ sex, age, and body mass index (BMI).

	Complete Structural Model			Refined Structural Model		
Independent Variable → Dependent Variable (Factor of Adjusted Measurement Model of Scale)	β	SE	*p*	β	SE	*p*
TEMS						
Sex → Health	−0.06	0.09	0.147	-	-	-
Age → Health	0.20	0.01	<0.001 *	0.20	0.01	<0.001 *
BMI → Health	−0.19	0.01	<0.001 *	−0.19	0.01	<0.001 *
Sex → Natural Concerns	−0.05	0.10	0.246	-	-	-
Age → Natural Concerns	0.22	0.01	<0.001 *	0.22	0.01	<0.001 *
BMI → Natural Concerns	−0.09	0.01	0.055	-	-	-
Sex → Socialization	−0.11	0.09	0.018 *	−0.11	0.09	0.022 *
Age → Socialization	−0.05	0.01	0.269	-	-	-
BMI → Socialization	0.03	0.01	0.444	-	-	-
Sex → Price	0.03	0.09	0.578	-	-	-
Age → Price	−0.02	0.01	0.592	-	-	-
BMI → Price	0.07	0.01	0.135	-	-	-
Sex → Visual Appeal	−0.05	0.09	0.260	-	-	-
Age → Visual Appeal	−0.05	0.01	0.303	-	-	-
BMI → Visual Appeal	0.05	0.01	0.302	-	-	-
Sex → Weight Control	0.03	0.09	0.490	-	-	-
Age → Weight Control	0.21	0.01	<0.001 *	0.22	0.01	<0.001 *
BMI → Weight Control	0.07	0.01	0.149	-	-	-
Sex → Emotional Control	−0.24	0.09	<0.001 *	−0.24	0.09	<0.001 *
Age → Emotional Control	−0.14	0.01	0.002 *	−0.14	0.01	0.002 *
BMI → Emotional Control	0.30	0.01	<0.001 *	0.30	0.01	<0.001 *
Sex → Social Image	−0.06	0.10	0.265	-	-	-
Age → Social Image	−0.06	0.01	0.212	-	-	-
BMI → Social Image	0.10	0.01	0.066	-	-	-
Adjustment indices of structural model	CFI = 0.95; TLI = 0.95; RMSEA = 0.05 (90% CI = 0.05–0.06)			CFI = 0.98; TLI=0.98; RMSEA = 0.05 (90% CI = 0.04–0.06)		

Source: Prepared by the authors, 2023. Note: β: standardized trajectory, SE: standard error of the trajectory, CFI: Comparative Fit Index, TLI: Tucker–Lewis Index, RMSEA: Root Mean Square Error of Approximation, 90% CI: 90% confidence interval. * statistical significance (*p* < 0.05).

## Data Availability

The questionnaire applied, as well as the informed consent form provided to the study participants, can be made available in full upon request to the corresponding author.

## Supplementary Materials

The following supporting information can be downloaded at: https://www.mdpi.com/article/10.3390/ijerph23010089/s1, Table S1: Psychometric sensitivity of data obtained from the Eating Motivation Survey (TEMS). Table S2: Distribution of responses to items in the Eating Motivation Survey (TEMS). Table S3: Correlation matrix between factors of the Eating Motivation Survey (TEMS).

## Abbreviations

The following abbreviations are used in this manuscript:

CFA	Confirmatory Factor Analysis
CAAE	Certificate of Submission for Ethical Assessment, the official acronym in Portuguese for “Certificado de Apresentação para Apreciação Ética”
CR	Composite Reliability
CFI	Comparative Fit Index
EATMOT	The Eating Motivations Scale
FCQ	Food Choice Questionnaire
df	Degrees of Freedom
HIV/AIDS	Human Immunodeficiency Virus/Acquired Immune Deficiency Syndrome
HTMT	Heterotrait–Monotrait ratio
90% CI	90% Confidence Interval
BMI	Body Mass Index
MI	Modification Indices
PLHIV	People Living with HIV
RMSEA	Root-Mean-Square Error of Approximation
SRMR	Standardized Root Mean Squared Residual
ICF	Informed Consent Form
TEMS	The Eating Motivation Survey
TLI	Tucker–Lewis Index
Ufac	Federal University of Acre
AVE	Average Variance Extracted
VIGITEL	Surveillance of Risk and Protective Factors for Chronic Diseases by Telephone Survey, acronym in Portuguese for “Vigilância de Fatores de Risco ou Proteção para Doenças Crônicas por Inquérito Telefônico”
WLSMV	Weighted Least Squares Mean and Variance Adjusted
χ2	Chi-square
α	Alpha (ordinal alpha coefficient)
β	Hypothetically causal paths (in Structural Equation Modeling)
λ	Factor loading
ω	Omega coefficient
