# Supplementary material for: Validity and Applicability of the Eating Motivation Survey (TEMS) in a University Population in the Western Brazilian Amazon
Source: Int J Environ Res Public Health. 2026 Jan 9;23(1):89. doi: 10.3390/ijerph23010089 (PMC12841441; doi:10.3390/ijerph23010089)
Supplement: Supplementary file 1 [file ijerph-23-00089-s001.zip › ijerph-3872541-supplementary.pdf]

SUPPLEMENTARY MATERIAL

Supplementary Table S1. Psychometric sensitivity of data obtained from *The Eating Motivation Survey* (TEMS).

Item	Responses<br><br>Why do you eat what you eat? | I eat what I eat...	Mean	Median	Mode	Standard Deviation	Skewness	Standard Error of Skewness	Kurtosis	Standard Error of Kurtosis	Minimum	Maximum
1	... because I am hungry.	3.8	4	4	0.9	-0.4	0.1	-0.2	0.2	1	5
2	... because it tastes good.	3.6	4	4	0.9	-0.1	0.1	-0.5	0.2	1	5
3	... because I feel like eating it.	3.5	4	3	1.0	-0.2	0.1	-0.5	0.2	1	5
4	... because it is quick to prepare.	3.2	3	3	1.1	0.0	0.1	-0.6	0.2	1	5
5	... because I am frustrated.	2.3	2	1	1.3	0.6	0.1	-0.7	0.2	1	5
6	... because it is low in calories.	2.2	2	1	1.1	0.7	0.1	-0.4	0.2	1	5
7	... because it is inexpensive.	3.3	3	3	1.1	-0.3	0.1	-0.5	0.2	1	5
8	... because it keeps me energized and motivated.	3.0	3	3	1.1	-0.1	0.1	-0.7	0.2	1	5
9	... because it is trendy.	1.4	1	1	0.6	2.0	0.1	5.0	0.2	1	5
10	... because I am familiar with the product.	3.5	4	4	1.1	-0.5	0.1	-0.3	0.2	1	5
11	... because it belongs to certain situations.	2.7	3	3	1.0	0.0	0.1	-0.4	0.2	1	5
12	... because I am sad.	2.4	2	1	1.2	0.5	0.1	-0.8	0.2	1	5
13	... because I control my weight.	2.3	2	1	1.3	0.6	0.1	-0.7	0.2	1	5
14	... for tradition (e.g., family traditions, special occasions).	2.5	3	3	1.1	0.3	0.1	-0.6	0.2	1	5
15	... because it is part of a social situation.	2.6	3	3	1.1	0.2	0.1	-0.5	0.2	1	5
16	... to treat myself to something really special.	2.7	3	3	1.1	0.3	0.1	-0.3	0.2	1	5

17	… to maintain a balanced diet.	2.9	3	3	1.2	0.1	0.1	-0.9	0.2	1	5
18	… because it satisfies my hunger in a pleasant way.	3.5	4	4	1.0	-0.4	0.1	-0.3	0.2	1	5
19	… because it would be impolite not to eat.	2.1	2	2	1.0	0.7	0.1	0.2	0.2	1	5
20	… because it is organic.	2.3	2	2	1.1	0.5	0.1	-0.5	0.2	1	5
21	… because it is convenient.	2.8	3	3	1.1	0.0	0.1	-0.8	0.2	1	5
22	… because it gives me pleasure.	3.3	3	3	1.1	-0.3	0.1	-0.5	0.2	1	5
23	… because I like it.	3.9	4	4	0.9	-0.6	0.1	0.1	0.2	1	5
24	… because its presentation is attractive (e.g., packaging).	2.6	3	2	1.1	0.3	0.1	-0.5	0.2	1	5
25	… to avoid disappointing someone who is trying to please me.	2.2	2	2	1.0	0.7	0.1	-0.1	0.2	1	5
26	… because it is natural.	3.0	3	3	1.1	0.0	0.1	-0.7	0.2	1	5
27	… so that I can spend time with other people.	2.5	3	3	1.1	0.3	0.1	-0.5	0.2	1	5
28	… because I do not want to spend too much money.	3.1	3	3	1.2	-0.1	0.1	-0.7	0.2	1	5
29	… because it does not contain harmful substances (e.g., pesticides, pollutants, antibiotics).	2.6	2	2	1.2	0.3	0.1	-0.8	0.2	1	5
30	… because it immediately catches my attention (e.g., in the supermarket, it is colorful).	2.2	2	2	1.0	0.5	0.1	-0.1	0.2	1	5
31	… because it gives me a good image in front of others.	1.6	1	1	0.9	1.5	0.1	1.9	0.2	1	5

32	… because I have to eat.	3.4	3	3	1.2	-0.3	0.1	-0.8	0.2	1	5
33	… because it is easy to prepare.	3.2	3	3	1.0	-0.2	0.1	-0.4	0.2	1	5
34	… because social gatherings become more enjoyable.	2.6	3	3	1.1	0.3	0.1	-0.6	0.2	1	5
35	… because I recognize it from advertisements or have seen it on TV.	1.9	2	2	0.9	0.7	0.1	0.2	0.2	1	5
36	… because I need energy.	3.4	4	4	1.1	-0.4	0.1	-0.5	0.2	1	5
37	… because I feel lonely.	2.0	2	1	1.2	0.9	0.1	-0.1	0.2	1	5
38	… because it is low in fat.	2.4	2	3	1.2	0.4	0.1	-0.6	0.2	1	5
39	… to reward myself.	2.8	3	3	1.1	0.1	0.1	-0.7	0.2	1	5
40	… because it is what I usually eat.	3.7	4	4	0.9	-0.7	0.1	0.4	0.2	1	5
41	… because it is on sale.	3.1	3	3	1.0	0.0	0.1	-0.4	0.2	1	5
42	… because others like it.	1.6	1	1	0.8	1.5	0.1	2.2	0.2	1	5
43	… because I grew up eating this way.	3.2	3	3	1.1	-0.2	0.1	-0.6	0.2	1	5
44	… because it is healthy.	3.2	3	3	1.1	-0.1	0.1	-0.6	0.2	1	5
45	… because I am used to eating it.	3.8	4	4	0.9	-0.5	0.1	0.0	0.2	1	5

Source: Prepared by the authors, 2023.

Supplementary Table S2. Distribution of responses to items in The Eating Motivation Survey (TEMS).

	Response options – n (%)				
**Item**	**1. Never**	**2. Rarely**	**3. Sometimes**	**4. Often**	**5. Always**
1	6 (0.9)	38 (6.0)	186 (29.4)	254 (40.2)	148 (23.4)
2	3 (0.5)	53 (8.4)	222 (35.1)	241 (38.1)	113 (17.9)
3	9 (1.4)	82 (13.0)	223 (35.3)	219 (34.7)	99 (15.7)
4	41 (6.5)	127 (20.1)	231 (36.6)	156 (24.7)	77 (12.2)
5	213 (33.7)	167 (26.4)	127 (20.1)	77 (12.2)	48 (7.6)
6	220 (34.8)	189 (29.9)	138 (21.8)	66 (10.4)	19 (3.0)
7	43 (6.8)	90 (14.2)	219 (34.7)	181 (28.6)	99 (28.6)
8	69 (10.9)	142 (22.5)	205 (32.4)	159 (25.2)	57 (9.0)
9	444 (70.3)	155 (24.5)	27 (4.3)	4 (0.6)	2 (0.3)
10	35 (5.5)	74 (11.7)	162 (25.6)	236 (37.3)	125 (19.8)
11	89 (14.1)	163 (25.8)	266 (42.1)	92 (14.6)	22 (3.5)
12	199 (31.5)	145 (22.9)	168 (26.6)	74 (11.7)	46 (7.3)
13	208 (32.9)	175 (27.7)	119 (18.8)	80 (12.7)	50 (7.9)
14	136 (21.5)	170 (26.9)	211 (33.4)	81 (12.8)	34 (5.4)
15	122 (19.3)	177 (28.0)	222 (35.1)	85 (13.4)	26 (4.1)
16	95 (15.0)	174 (27.5)	250 (39.6)	74 (11.7)	39 (6.2)
17	89 (14.1)	148 (23.4)	188 (29.7)	130 (20.6)	77 (12.2)
18	24 (3.8)	82 (13.0)	192 (30.4)	234 (37.0)	100 (15.8)
19	207 (32.8)	231 (36.6)	146 (23.1)	34 (5.4)	14 (2.2)
20	175 (27.7)	206 (32.6)	151 (23.9)	79 (12.5)	21 (3.3)
21	100 (15.8)	152 (24.1)	205 (32.4)	140 (22.2)	35 (5.5)
22	45 (7.1)	87 (13.8)	209 (33.1)	195 (30.9)	96 (15.2)
23	5 (0.8)	32 (5.1)	138 (21.8)	273 (43.2)	184 (29.1)
24	114 (18.0)	200 (31.6)	199 (31.5)	85 (13.4)	34 (5.4)
25	197 (31.2)	223 (35.3)	144 (22.8)	50 (7.9)	18 (2.8)
26	65 (10.3)	144 (22.8)	218 (34.5)	143 (22.6)	62 (9.8)
27	124 (19.6)	182 (28.8)	215 (34.0)	84 (13.3)	27 (4.3)
28	70 (11.1)	113 (17.9)	214 (33.9)	152 (24.1)	83 (13.1)
29	139 (22.0)	183 (29.0)	170 (26.9)	98 (15.5)	42 (6.6)
30	171 (27.1)	225 (35.6)	177 (28.0)	44 (7.0)	15 (2.4)
31	365 (57.8)	166 (26.3)	76 (12.0)	17 (2.7)	8 (1.3)
32	49 (7.8)	92 (14.6)	190 (30.1)	162 (25.6)	139 (22.0)
33	44 (7.0)	105 (16.6)	235 (37.2)	189 (29.9)	59 (9.3)
34	121 (19.1)	187 (29.6)	200 (31.6)	96 (15.2)	28 (4.4)
35	233 (36.9)	245 (38.8)	131 (20.7)	18 (2.8)	5 (0.8)
36	34 (5.4)	86 (13.6)	193 (30.5)	205 (32.4)	114 (18.0)
37	286 (45.3)	154 (24.4)	102 (16.1)	57 (9.0)	33 (5.2)
38	168 (26.6)	173 (27.4)	179 (28.3)	78 (12.3)	34 (5.4)
39	105 (16.6)	139 (22.0)	227 (35.9)	115 (18.2)	46 (7.3)
40	16 (2.5)	41 (6.5)	162 (25.6)	289 (45.7)	124 (19.6)
41	41 (6.5)	139 (22.0)	255 (40.3)	138 (21.8)	59 (9.3)
42	359 (56.8)	198 (31.3)	57 (9.0)	14 (2.2)	4 (0.6)
43	52 (8.2)	107 (16.9)	222 (35.1)	167 (26.4)	84 (13.3)
44	44 (7.0)	104 (16.5)	235 (37.2)	158 (25.0)	91 (14.4)
45	6 (0.9)	37 (5.9)	164 (25.9)	281 (44.5)	144 (22.8)

Source: Prepared by the authors. 2023.

Supplementary Table S3. Correlation matrix between factors of *The Eating Motivation Survey* (TEMS)

	A	B	C	D	E	F	G	H	I	J	K	L	M	N	O
**A. Preference**	1	0.47*	0.57*	0.09	0.26*	0.82*	0.32*	0.07	0.25*	0.12*	0.39*	-0.02	0.30*	0.09	0.11
**B. Habits**	-	1	0.85*	0.28*	0.49*	0.40*	0.96*	0.30*	0.32*	0.40*	0.22*	0.18*	0.04	0.35*	0.09
**C. Need and Hunger**	-	-	1	0.93*	0.45*	0.55*	0.69*	0.69*	0.40*	0.32*	0.25*	0.52*	-0.11	0.39*	0.20*
**D. Health**	-	-	-	1	-0.05	0.07	0.26*	0.85*	0.18*	-0.07	0	0.81*	-0.27*	0.09	0.10
**E. Convenience**	-	-	-	-	1	0.39*	0.66*	0.07	0.52*	0.67*	0.42*	0	0.34*	0.52*	0.47*
**F. Pleasure**	-	-	-	-	-	1	0.57*	0.12*	0.51*	0.27*	0.56*	0.07	0.69*	0.29*	0.42*
**G. Traditional Food**	-	-	-	-	-	-	1	0.37*	1.00*	0.50*	0.56*	0.37*	0.56*	0.81*	0.64*
**H. Natural Questions**	-	-	-	0.86*	-	-	-	1	0.29*	0.07	0.15*	0.74*	-0.09	0.20*	0.22*
**I. Socialization**	-	-	-	0.18*	-	-	-	0.29*	1	0.37*	0.59*	0.27*	0.41*	0.75*	0.70*
**J. Price**	-	-	-	-0.08	-	-	-	0.07	0.37*	1	0.34*	0.01	0.36*	0.37*	0.35*
**K. Visual Attraction**	-	-	-	0	-	-	-	0.15*	0.59*	0.34*	1	0.01	0.48*	0.53*	0.74*
**L. Weight Control**	-	-	-	0.81*	-	-	-	0.75*	0.27*	0.01	0.06	1	0.02	0.21*	0.35*
**M. Emotion Control**	-	-	-	-0.27*	-	-	-	-0.09*	0.41*	0.36*	0.48*	0.02	1	0.36*	0.60*
**N. Social Norms**	-	-	-	-	-	-	-	-	-	-	-	-	-	1	0.80*
**O. Social Image**	-	-	-	0.10	-	-	-	0.23*	0.70*	0.35*	0.74*	0.35*	0.60*	-	1

Note: Values below diagonal 1 are from the refined and adjusted TEMS model for the sample of the present study, and values above diagonal 1 are from the original model.
* indicates statistical significance (p<0.05).
